# Renal manifestations of sarcoidosis: from accurate diagnosis to specific treatment

**DOI:** 10.1590/S1677-5538.IBJU.2019.0042

**Published:** 2020-01-13

**Authors:** Filipe A. Saliba C. Correia, Giovanni S. Marchini, Fábio C. Torricelli, Alexandre Danilovic, Fábio C. Vicentini, Miguel Srougi, William C. Nahas, Eduardo Mazzucchi

**Affiliations:** 1 Seção de Endourologia, Divisão de Urologia - Hospital das Clínicas da Faculdade de Medicina da Universidade de São Paulo, SP, Brasil

**Keywords:** Sarcoidosis, therapy [Subheading], Granulomatous Disease, Chronic

## Abstract

Sarcoidosis is a multisystem granulomatous disease characterized by epithelioid noncaseating granulomas associated with clinical and radiologic findings. The cause of this disease is still uncertain. Sarcoidosis affects mostly lungs and lymph nodes and is not usually considered a urological disease, therefore, this etiology may be overlooked in several urological disorders, such as hypercalcemia, hypercalciuria and nephrolithiasis. It affects all races and genders. This review aims to describe the urological manifestations of sarcoidosis and to elucidate how the disease may affect the management of numerous urological conditions.

## INTRODUCTION

Sarcoidosis is a multisystem granulomatous disease characterized by non-caseating epithelioid granulomas in association with clinical and radiologic findings. The cause of this disease is still uncertain. Recent findings suggest that sarcoidosis is related to a chronic immune response caused by exposure to common environmental factors such as Propionibacterium or airborne organic or inorganic material (1), most probably a sum of several immune system and environmental factors (2). It affects all races and genders; however, women are 30% more likely to be affected than men and African-Americans (36/100.000) are more commonly affected than Caucasians (11/100.000) (3). In Europe the incidence is higher in northern countries, 20-40/100.000 at general, up to 121/100.00 in Sweden and lower in southern countries like England (5/100.000) and Spain (1.36/100.000) (4). Japan has a reported prevalence of 0.3-1.7/100.000 (5). Brazil does not have recent prevalence studies; the only one was published in 1985 with an estimative of 10 cases per 100.000 inhabitants (4). Genetic propensity may explain the heterogeneity at appearance and the severity of the cases in different ethnic groups and races (2).

Patients with sarcoidosis usually presents with symptoms before the age of 50, with a peak between 20-39 years old. Suggestive findings on chest x-ray of asymptomatic patients are also another form of diagnosis. However, cough, shortness of breath, fatigue or night sweats may be present (6). Most patients with sarcoidosis present one of the following: intrathoracic lymphadenopathy, pulmonary involvement, cutaneous symptoms or eye impairment. Skin manifestations include macules, papules, simple or multiple plaques, which can commonly affect the face, posterior neck, torso and extremities. Erythema nodosum may be present transitorily in 10% of the patients, most commonly in women (7). Most patients with sarcoidosis will experience remission of the disease and will never require specific treatment. However, a third will experience chronic potentially severe disease and ultimately the specific mortality rate may be up to 5% (1). Treatment is mainly based on corticosteroids or immunosuppressive agents to control symptoms.

Sarcoidosis is not usually considered a urological disease, affecting mostly lungs and lymph nodes. For that reason, it may be overlooked when it affects the urinary tract. However, urinary impairment of the disease is not rare and may lead to conditions treated by the urologist such as nephrolithiasis. Moreover, the disease may also produce clinical manifestations that can mimic severe urological disorders such as testicular nodules, renal masses, or even PET positive lymphadenopathy, leading to misinterpretations of early stage urological malignancies (6). The aim of this study is to review how sarcoidosis may affect and interact with several urological illnesses and to describe how to perform an accurate diagnosis and a patient-centered approach.

## MATERIALS AND METHODS

An online review was done searching for urological conditions and manifestations associated with sarcoidosis. A research was performed using the key words “sarcoidosis” combined with the urological terms “calculus”, “calculi”, “nephrolithiasis”, “hypercalciuria”, “kidney”, “renal” and “urinary” published until June 2017 in PubMed and Google Scholar database. The results of more than 1.000 articles were summed up to 80 articles and all the relevant information was gathered, organized, and brought to discussion, in addition, the significant references quoted in the selected articles where added to the research.

Two separate urologists performed the online search and reviewed all papers considered suitable and relevant for this analysis. Because of the paucity of high-quality publications, not only prospective and review papers but also case control and case series studies were included in the final analysis. After extensive evaluation and analysis of the data, the information regarding urological manifestations of Sarcoidosis was divided in specific sessions to facilitate and summarize the findings: hypercalcemia and hypercalciuria; nephrolithiasis and nephrocalcinosis, granulomatous interstitial nephritis, glomerular disease and tubular dysfunction, diagnosis and treatment.

## RESULTS

Sarcoidosis has a wide range of renal manifestations, most of them related to calcium metabolism, which may ultimately cause renal dysfunction. The focus of our review is the urological manifestations of the disease that may also coexist.

### Hypercalcemia and Hypercalciuria

The calcium metabolism disorders occur in patients with sarcoidosis and are presented by hypercalcemia or hypercalciuria due to activated macrophages expressing 1alpha-hydroxilase in sarcoid granulomas (8), this leads to increased levels of 1.25 dihydroxy vitamin D (calcitriol), resulting in high calcium absorption from the bowels (9). Hypercalcemia is present in 10 to 17% of patients with sarcoidosis (10). An altered level of vitamin D and hypercalcemia causes a suppression of parathyroid hormone (10). The suppressed PTH and this overloaded blood calcium is urine excreted, causing hypercalciuria − 24-hour urinary levels of calcium above 300mg/dL. Hypercalciuria may be found in 2-5% in healthy adults and up to 62% (11) in patients with sarcoidosis, and a more severe state occurs in 10-20% (6). Excessive sunlight or vitamin D ingestion may worsen the case. These parameters may change according to disease activity or a patient's total ultra-violet light exposure (12). Hypercalciuria predisposes to nephrolithiasis and obstructive uropathy (13).

Hypercalcemia is responsible for a decrease in glomerular filtration rate by vasoconstriction of the afferent arteriole. Also, it inhibits sodium-potassium ATPase leading to urinary sodium wasting with polyuria and dehydration. Finally, a reduced sensitivity to anti-diuretic hormone impairs urinary concentration. Acute tubular necrosis may occur due to intracellular calcium overload and tubular obstruction by calcium precipitates. Hypercalciuria ultimately leads to nephrolithiasis. In the acute phase, the consequences of hypercalcemia and hypercalciuria are reversible but once fibrosis takes place, the damage becomes irreversible (12, 14).

### Nephrolithiasis and Nephrocalcinosis

**Figure 1:**
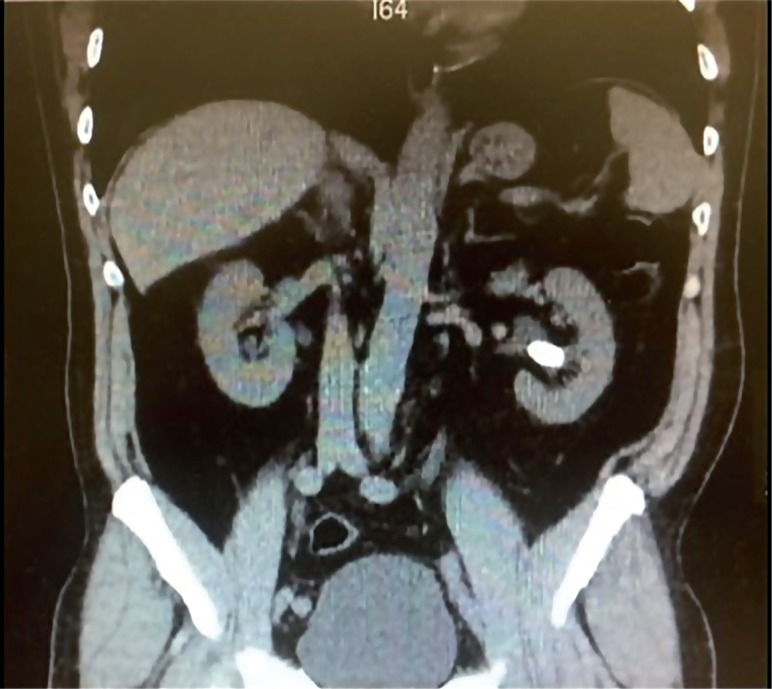
Nephrolitiasis.

Nephrolithiasis (Figure-1) has been found in about 10% of patients with sarcoidosis, with a prevalence range of 3% to 14%, (14) and hypercalciuria may not be present in all cases at the moment of the diagnosis or when the calculus becomes symptomatic (9). Nephrolithiasis has been referred as the first sign for the diagnosis of sarcoidosis in some patients (11–17). In studies with a careful review of patient's medical history, renal colic was the first sign of the disease in 2.2% of cases (10). However, the diagnosis was done in only half of the patients by the time of the renal calculi diagnosis. The other half of patients had their diagnosis only when other symptoms of chronic sarcoidosis became clinically significant. In these studies, most patients with nephrolithiasis had pulmonary involvement on chest x-ray and the majority also had palpable lymphadenopathy or cutaneous lesions. This combination of findings should alarm the urologist and patients presenting with calcium-based kidney stones that otherwise would be in a low-risk group for nephrolithiasis, such as African American females, who should undergo a lymphadenopathy and skin physical examination and chest x-ray for signs of sarcoidosis.

**Figure 2:**
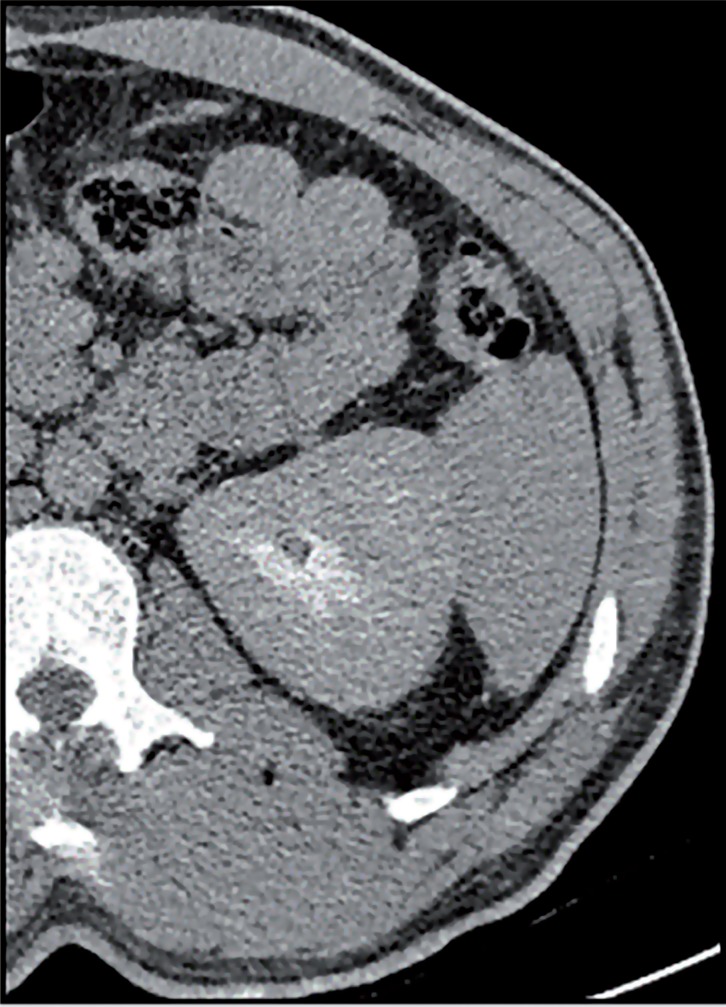
Nephrocalcinosis.

Nephrocalcinosis (Figure-2) is a rare impairment and is a result of chronic hypercalciuria (16, 17). It is present in less than 5% of patients with sarcoidosis but in a higher rate of patients with renal insufficiency (9). It is a condition related to the calcification of the renal parenchyma and tubules frequently associated with sarcoidosis among other disorders. Advanced macroscopic disease often diagnosed by imaging studies in individuals suspected to have clinical and laboratory findings of sarcoidosis, however, usually is an incidental finding in asymptomatic patients with otherwise unremarkable laboratory parameters. The diagnosis of the initial stages of nephrocalcinosis can be exposed through renal biopsy demonstrating calcium deposits of either calcium phosphate or calcium oxalate on analysis (18). Ultrasound (US) and Computed Tomography (CT) are the preferred diagnostic modalities for nephrocalcinosis as they have sensitivity values of 85 to 90% and 81 to 86%, respectively, with more specificity for the CT − 83% to 89% then the US − 66% to 71% (19). Renal studies with biopsy demonstrated renal sarcoid infiltration in 7-40%, most likely the epithelioid granuloma (11), and it was found up to 20% patients affected in post-mortem dissection (20). These cases might present with sterile leukocyturia, hematuria and proteinuria. In present times, renal granulomas are rare thought when present may simulate kidney neoplasia (2).

### Granulomatous interstitial nephritis

It is the most common renal lesion seen on biopsy. The true incidence of granulomatous interstitial nephritis is unknown. In autopsy studies, granulomatous infiltrate in kidney tissue was found in up to 23% and in small case series of renal biopsy the incidence of granulomatous interstitial nephritis was 48%, most of them clinically silent (13).

The urinary manifestations of interstitial nephritis are like those of other tubule-interstitial diseases. Urinalysis findings will be most commonly normal, however, these may show sterile pyuria, microscopic hematuria, glycosuria, and hypercalciuria (21, 22).

### Glomerular disease and tubular dysfunction

A variety of lesions including membranous nephropathy, focal segmental sclerosis, mesangioproliferative glomerulonephritis, IgA nephropathy and crescent glomerulonephritis are described as glomerular involvement, indistinguishable from the primary form. Tubular dysfunctions can be present in isolated proximal or distal renal tubular acidosis or Fanconi's syndrome. Polyuria is the usual clinical feature, mostly because of hypercalcemia disorders (18).

### Diagnosis

Sarcoidosis is a diagnosis of exclusion; almost half of the patients takes more than 3 physician visits until the diagnosis is made (23). But up to 90% of the times an altered x-ray of the chest starts the investigation (24), even for patients with no respiratory symptoms (i.e. preoperative exams). Some of the classic x-ray findings are described in the Scadding scale but most of all abnormal x-ray findings can relate to sarcoidosis. The clinical presentation is a wide variety of symptoms. Pulmonary cough, dyspnea, shortness of breath, fatigue or night sweats (25). Lymph nodes, mainly hilar adenopathy (95-98%) and also peripheral lymph nodes (10-20%). Cutaneous manifestations are highly prevalent (15%) more commonly erythema nodosum and lupus pernio. Association with uveitis (10-30%) and neuropathy of the facial nerve (5%) are highly suggestive findings (7). Nephrolithiasis and nephrocalcinosis are more prevalent in these patients and the evidence of hypercalcemia (10-17%) e hypercalciuria (67%) are common symptoms and belong to daily practice of the urologist (10, 25, 26).

In the other hand, sarcoidosis is a multiorgan disease and a patient with no pulmonary or lymph nodes may still have sarcoidosis. 10% of patients may not present any of those at first (25), and urological findings have been referred as the first sign for the diagnosis of sarcoidosis in some and renal colic was the first sign of the disease in 2.2% of cases (10). For this, the urologist should always have in the follow-up of his patients the possibility of sarcoidosis in mind and investigate if some other findings start to present, due to a slow onset of symptoms.

In an appropriate clinical setting, the presence of non-necrotizing granulomas with no evidence of infection is the usual criterion to suggest the diagnosis. Sarcoidosis mimics nonspecific granulomatous reactions. These should be excluded by a careful examination and by medical, occupational, and medication histories. In practice, the disease is most often diagnosed by biopsy of accessible tissues, usually skin, lungs, or peripheral lymph nodes (27). Sarcoidosis is a systemic granulomatous disease and the diagnosis usually requires the demonstration of typical lesions in more than one organ system and it is recommended a histologic diagnosis before commencing any treatment (26). The disease manifestation in several systems and incidence are exposed on Table-1.

**Table 1:** Extra pulmonary signs and symptoms of Sarcoidosis.

Organ/System	Prevalence (%)	Simptoms	Investigation
Cutaneous system	15% (9-37%)	Papules, nodules, plaques, scar sarcoidosis, lupus pernio, subcutaneous sarcoidosis	Biopsy
Peripheral lynphnodes	10-20%	Mostly cervical or supraclavicular, inguinal, axillar, epitrochlear or submandibular lymph node sites; painless and mobile	Biopsy
Ocular system	10-30%	Anterior, intermediate, or posterior uveitis, retinal vascular change, conjuntival nodules, lacrimal gland enlargement	Systematics ophtalmologist exam, slit-lamp exam, fluorescein angiography
Hepatic (Gastrointestinal system)	20-30%	Often symptom-free. Abnormal liver function test, hepatomegaly, rarely cholestasis, portal hypertension, hepatic insufficiency	Systematics liver function tests, CT, biopsy
Splenic (Gastrointestinal system)	10%	Splenomegaly; rarely pain, pancytopenia; very rarely, splenic rupture	Echography, CT
Cardiovascular system	2-5%	Atrioventricular or bundle branch block, ventricular tachycardia or fibrillation, congestive heart failure, pericarditis, irmpaiment of sympathetic nerve activity	Eletrocardiography, Echocardiography, BNP, MRI, scintigraphy, FDG PET
Nervous System	5%	Facial nerve palsy, optic neuritis, leptomeningitis, diabetis insipidus, hypopituitarism, seizures, cognitive dysfunction, deficts, hydrocefalus, psychiatric manifestations, spinal cord disease	Cerebrospinal fluid investigation, MRI, hormonal dose, eletromyography, biopsya rarely done
Renal system	5-20%	Increased creatininemia, hypercalcemia, nephrocalcinosis, kidney stones	Systematic renal tests, biopsy

Extrapulmonary investigation Adapted - Sarcoidosis(25); Management of extrapulmonary sarcoidosis (27).

Renal sarcoidosis is often accompanied by systemic manifestations although isolated renal sarcoidosis is an accepted entity. The presence of sarcoid-related granulomatous interstitial nephritis is the goal but as related before, the absence of characteristic kidney biopsy findings does not exclude the diagnosis and clinical manifestations with other tissues biopsy should be attempted (13). All patients diagnosed with sarcoidosis should be evaluated for the presence of renal involvement to prevent significant chronic kidney disease (28).

Bronchoscopy is a reliable, minimally invasive technique to diagnose the disease. Combination of transbronchial biopsy with endobronchial (mucosal) biopsy or transbronchial needle aspiration of enlarged lymph nodes increases the sensitivity of the technique to as much as 91%. Endobronchial ultrasonography (with ROSE - Rapid on-site cytological examinations) has been used more recently to further improve diagnostic yield. Samples should be analyzed for infectious agents by appropriate stains, including stains for mycobacteria and fungus, as well as by culture (27).

**Figure 3:**
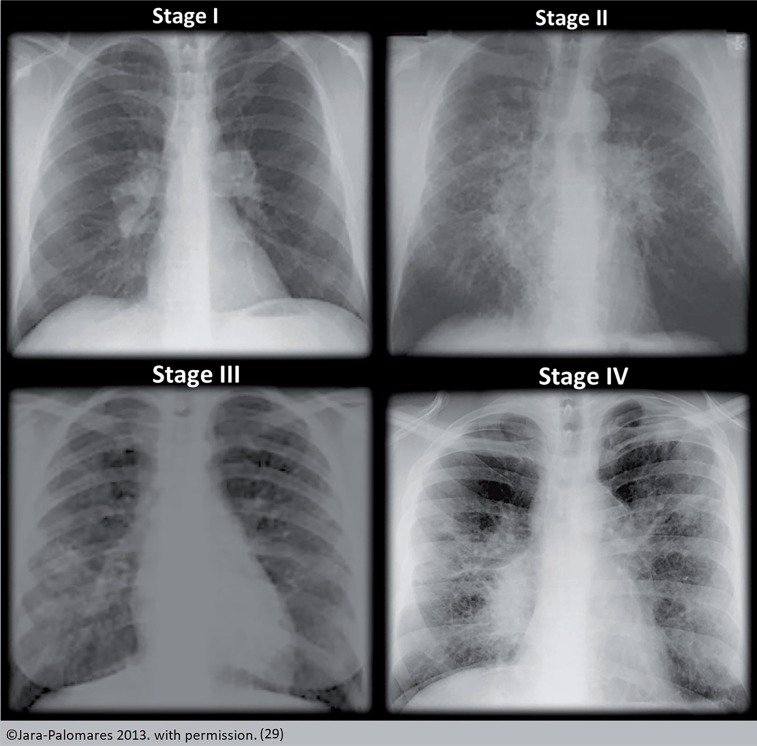
Scadding scale.

The Scadding scale, a descriptive schema is widely used to describe chest x-ray finding. It does not represent sequential or temporal disease states, and the predictive ability of the scale allows an approximation of outcomes. It was only described in the 19th century and standardized by the WASOG committee. Types are: 0 (normal radiological findings); I (bilateral mediastinal hilar adenopathy); II (adenopathy and pulmonary infiltrates); III (pulmonary infiltrates only); and IV (pulmonary fibrosis) (25). Features that should prompt consideration of an alternative diagnosis include pleural effusion, unilateral abnormalities, and the presence of calcification in the lymph nodes. Chest CT usually shows typical micronodular infiltrates distributed in a bronchial vascular pattern, often predominating in the mid to upper lung zones (13) (Figure-3).

Other diagnostic modalities for different organs testing may be appropriate and guided by the initial symptoms and examination findings. Routine testing for elevated liver enzymes, hypercalcemia, renal dysfunction, ophthalmologic involvement, and electrocardiographic abnormalities is standard at baseline. Magnetic resonance imaging (MRI) with gadolinium or gallium is useful for diagnosing neurologic involvement. Lumbar puncture should be performed in the appropriate clinical context to exclude mycobacterial or fungal infections. Thallium or sesamoid scintigraphy is useful to identify areas of active or inactive myocardial involvement. Newer imaging techniques, such as fluorodeoxyglucose positron emission tomography (FDG-PET) and gadolinium-enhanced MRI are promising modalities for diagnosis and for monitoring treatment response (27).

**Figure 4:**
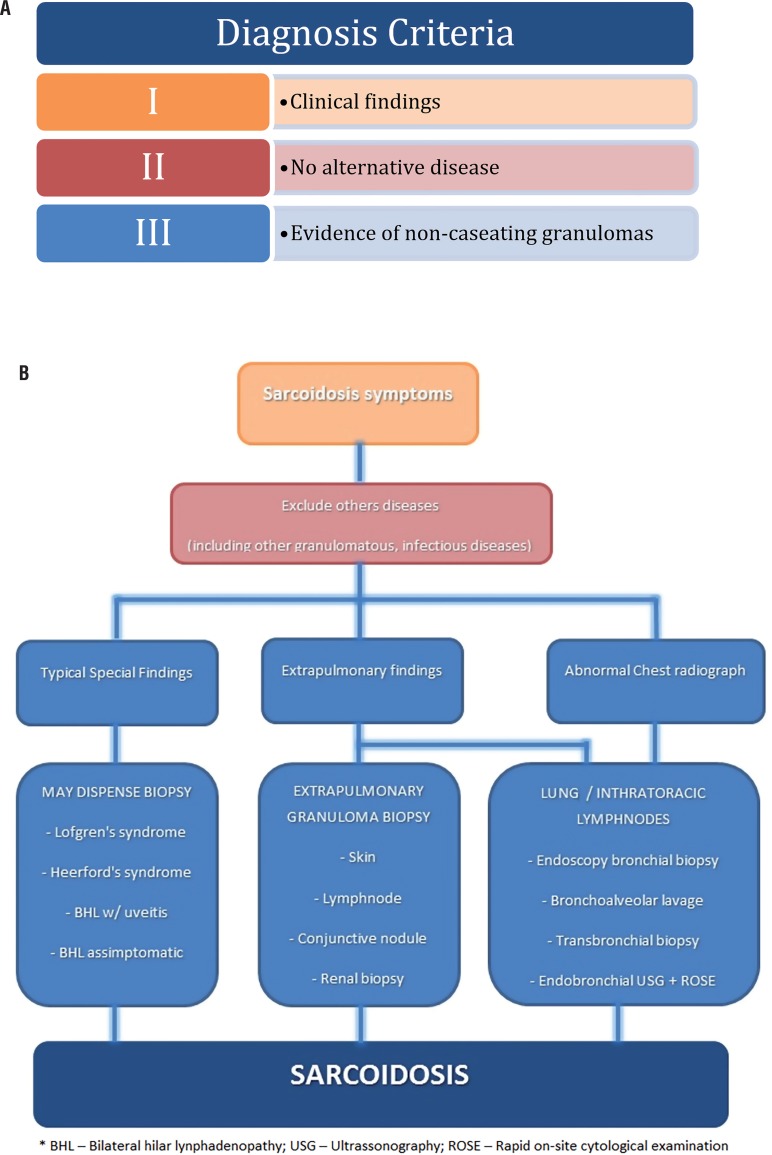
Algorithm for diagnosis of Sarcoidosis considering disease criteria (A) and rationale flow chart (B).

High levels of angiotensin converting enzyme (ACE) have been reported in sarcoidosis, the frequency varies from 40% up to 90% (2). The enzyme is produced by epithelioid cells, multinucleated giant cells, and macrophages within granulomas (13). It presents a false-negative rate of 40% and false-positive rate of 10%. Other conditions may raise the levels of ACE and might have a differential diagnosis with sarcoidosis such as Leprosy, Myeloma, Gaucher Disease, Amyloidosis, Acute Histoplasmosis, Hyperthyroidism, Hyperparathyroidism, Alcoholic Cirrhosis, Primary Biliary Cirrhosis, Oncogenic Hypercalcemic, Military Tuberculosis, Pulmonary Endothelia Disease, Elverson-Rosenthal syndrome - “Sarcoid-like” granuloma. Therefore, it must be analyzed with criteria but usually consists in more granulomatous impairment, pulmonary or systemic, and might be useful for treatment monitoring as the levels tend to normalize with the spontaneous or corticoid induced remission of the disease (2). Recent data have demonstrated that the presence of certain human leukocyte antigen (HLA) haplotypes (e.g., HLA-DR17 and HLA-DQB1) confers good prognosis in certain European populations (30). However, the usefulness of HLA typing in other populations has not been confirmed (27). A diagnosis algorithm is depicted on Figures 4a and 4b.

### Treatment

The initial treatment of sarcoidosis is glucocorticoids, the most effective, rapid-acting and available drug. It's the first line of treatment (31). The therapy significantly improves the clinical manifestations of sarcoidosis and normalizes hypercalcemia and hypercalciuria associated with this disease. Corticosteroids down regulate the activity of 1alphahidroxylase in the pulmonary macrophages and granulomas normalizing the blood and urinary levels of calcium (21). Hypercalciuria can lead to renal failure and the therapy improves renal function (9, 10). There is no standard dose or time for the treatment. It usually starts with low-dose prednisone 20-40mg/day for mild disease or every other day. For major organ impairment, a dose of 1mg/kg/d is usually used. The follow-up is after 1-3 months with the response based on clinical progress, pulmonary radiology and functional status, tapering up to 5mg/week after the initial period (13, 31). Corticosteroids have many well-known side effects, the most important being diabetes, hypertension, osteoporosis and central obesity (32). The medical management of sarcoidosis according to clinical manifestation is resumed on Table-2.

**Table 2:** Medical management of Sarcoidosis according to clinical manifestation.

Renal Manifestations	Initial Treatment	Alternative Treatment	Alt. Treatment	Alt. Treatment	Comments
Hypercalcemia Hypercalciuria	Glucocorticoids Initial: 0,3-0,5mg/ kg/d Mainteance: 5-10mg/d	Hydroxychloroquine 200-400mg/day	Ketoconazol 200-800mg/day		IV hydration Limit sunlight Low intake calcium, vitD and oxalate Avoid thiazide
Granulomatous Interstitial Nephritis - GIN	Glucocorticoids Major: 1mg/kg/d Mild: 0,5mg/kg/d Mainteance: 5-10mg/d	Azathioprine 2mg/kg/day (50-200mg/d)	Mycophenolate mofetil 1g twice a day (500-3000mg/d)	Infliximab 3-5mg/kg week 0, 2, 6 for 4-8weeks	Add a steroid-sparing agent to the threatment if relapse or dificulty to taper
Glomerular Disease	Glucocorticoids Initial: 1mg/kg/d Mainteance: 5-10mg/d	After GIN alternatives	Methotrexate 10-20mg/week		Folic acid supplementation
Tubular Dysfunction	Glucocorticoids Initial: 1mg/kg/d Mainteance: 5-10mg/d	After GIN alternatives	Methotrexate 10-20mg/week		Folic acid supplementation
Nephrolitiasis	Metabolic control	Surgical threatment of lithiasis			Hypercalcemia and hypercalciuria control
Nephrocalcinosis	Metabolic control				Hypercalcemia and hypercalciuria control Higher rate of renal failure
Treatment					

There are no major marks of the disease but ACE (angiotensin-converting enzyme) can be related to disease activity. Usually the treatment is due to 12 months. For advanced cases with no response to corticoids, other cytotoxic drugs may be used such as methotrexate and azathioprine (26). New immunomodulator drugs are being tested for specific cases and study protocols like infliximab and etanercept are on course. Non-responding cases with high calcium levels may benefit with ketoconazole use. In addition, a low calcium diet, adequate hydration, and avoiding exposure to sunlight may prevent deterioration of hypercalcemia and hypercalciuria. Supplements of vitamin D and calcium and calcium rich foods should be avoided (11).

Although not all cases of sarcoidosis present with hypercalcemia, the majority will have this electrolyte disorder. Acute symptomatic hypercalcemia is normally treated in hospital settings with intravenous infusion of normal saline solutions. Loop diuretics need to be added to facilitate urinary calcium excretion via the thick segment of the loop of Henle. Calcitonin is rarely used for acute hypercalcemia owing to its short acting effect on extracellular calcium levels. The use of glucocorticoids is an essential step for the treatment of hypercalcemia related to sarcoidosis as they suppress intestinal absorption of calcium and 1-alfa hydroxylase in sarcoid granulomas (19). Most authors recommend a starting dose of 0.3-0.5mg/kg/day and a maintenance dose of 5-10mg/day with duration of treatment of 12 months (13). Chloroquine with a dose of 200-400mg can be used as an alternative treatment for hypercalcemia and for patients with sarcoidosis who cannot be treated with glucocorticoids. Ketoconazole 200-800mg a day can be utilized as another non-prednisone alternative for the treatment of hypercalcemia (13). The mechanism of action for both drugs is related to the suppression of 1.25 dihydroxy vitamin D production in granulomas.

The use of bisphosphonate for sarcoidosis-related hypercalcemia with elevated plasma 1.25 dihydroxy vitamin D levels was reported to provide a rapid correction of plasma calcium levels without affecting vitamin D concentrations. One has to realize that the use of bisphosphonates will alleviate hypercalcemia, however, it will not influence the disease progression. Evaluation of a 24-h urine collection for calcium excretion is recommended in all patients with sarcoidosis (31). Moreover, learning about the complete metabolic profile of the urine may provide valuable insights in the management of nephrolithiasis related to hypercalcemia and hypercalciuria in these patients. Low dietary intake of calcium, vitamin D, oxalate and avoidance of thiazide-like diuretics (calcium-sparing proprieties) are generally recommended for patients with hypercalcemia. Limiting sunlight exposure is also advised to prevent the enhancement of vitamin D production (13).

Azathioprine - Immunosuppressive drug which can be used as steroid-sparing agent or in patients with failure or a contraindication to corticosteroids. It has a delayed effect, treatment with these drugs should start only after at least 1 month of treatment with glucocorticoids (13). Azathioprine should be given in a daily dose of 2mg/kg (50-200mg/day) and can be used in men and women who want to have children and used during pregnancy (31).

Methotrexate - It is an important agent and a preferred second-line in the treatment of extra-renal sarcoidosis where it can be used as an alternative to corticosteroids or as a steroid-sparing agent (13). Usual dose is of 10-20mg/week orally or intramuscularly (31). The renal excretion makes it not recommended for possible accumulation of the drug and development of major side effects.

Mycophenolate mofetil - Another immunosuppressive for steroid-sparing, with insufficient data but in theory leads to fewer bone marrow toxic effects and less infections than other immunosuppressive agents. The dose starts with 1000mg twice a day (500-3000mg/day) (31). It can improve kidney function previously damaged by the sarcoidosis (13).

TNF-alpha inhibitors - Treatment strategy after steroid-resistant/refractory disease. It should only be used after immunosuppressive agents have been tried (13). Infliximab dose starts from 3-5mg/kg at week 0, 2, 6 and then every 4-8 weeks (31). There is a rapid effect, as early as two weeks and steady improvement of renal function was reported (13). Possible loss of response due to anti-infliximab antibody formation.

Kidney transplantation End-stage renal disease secondary to sarcoidosis is uncommon. The disease generally occurs in young and middle-aged adults. Renal transplantation can be carried out safely in patients with sarcoidosis with excellent graft and patient survival (33). However, a relatively high rate of renal recurrence (17%) after transplantation was reported in most cases occurring shortly after transplantation with negative effect on graft function. The long-term effects of recurrence on graft survival remain elusive. A short delay between the last episode of sarcoidosis and renal transplantation is a risk factor for recurrence as is necessary in other diseases. Relapse after transplantation has been managed with infliximab in a steroid-resistant case (34).

## CONCLUSION

Sarcoidosis is considered an “iceberg” disease, from one initial symptom, the physician finds a complete pathology with multiples systems affected. Disordered calcium metabolism and renal involvement should always require treatment given the risk of the development of complications such polyuria, dehydration, kidney stones and even renal failure.
